# Transformer Models in Healthcare: A Survey and Thematic Analysis of Potentials, Shortcomings and Risks

**DOI:** 10.1007/s10916-024-02043-5

**Published:** 2024-02-17

**Authors:** Kerstin Denecke, Richard May, Octavio Rivera-Romero

**Affiliations:** 1 Institute Patient-centered Digital Health, Bern University of Applied Sciences, Quellgasse 21, Biel, 2502 Switzerland; 2 Harz University of Applied Sciences, Friedrichstraße 57-59, 38855 Wernigerode, Germany; 3 Instituto de Ingeniería Informática (I3US), Universidad de Sevilla, Sevilla, Spain; 4 Department of Electronic Technology, Universidad de Sevilla, Avda Reina Mercedes s/n, ETSI Informática, G1.43, Sevilla, 41012 Spain

**Keywords:** Large Language Model, Transformer Models, Artificial Intelligence, Healthcare, Generative Artificial Intelligence

## Abstract

Large Language Models (LLMs) such as General Pretrained Transformer (GPT) and Bidirectional Encoder Representations from Transformers (BERT), which use transformer model architectures, have significantly advanced artificial intelligence and natural language processing. Recognized for their ability to capture associative relationships between words based on shared context, these models are poised to transform healthcare by improving diagnostic accuracy, tailoring treatment plans, and predicting patient outcomes. However, there are multiple risks and potentially unintended consequences associated with their use in healthcare applications. This study, conducted with 28 participants using a qualitative approach, explores the benefits, shortcomings, and risks of using transformer models in healthcare. It analyses responses to seven open-ended questions using a simplified thematic analysis. Our research reveals seven benefits, including improved operational efficiency, optimized processes and refined clinical documentation. Despite these benefits, there are significant concerns about the introduction of bias, auditability issues and privacy risks. Challenges include the need for specialized expertise, the emergence of ethical dilemmas and the potential reduction in the human element of patient care. For the medical profession, risks include the impact on employment, changes in the patient-doctor dynamic, and the need for extensive training in both system operation and data interpretation.

**Supplementary Information:**

The online version contains supplementary material available at 10.1007/s10916-024-02043-5.

## Introduction

Rapid advances in artificial intelligence (AI) technologies, including large language models (LLMs) and generative AI, have created new opportunities and challenges for healthcare. An LLM is a machine learning model that encodes complex patterns of language usage derived from large amounts of input text. LLMs can use neural network architectures, typically enhanced with a transformer attention mechanism that capture associative relationships between words based on shared context. These transformer models were first introduced in 2017 by Vaswani et al. [1] and have already significantly changed the landscape of natural language processing (NLP). Originally developed for language-related applications, transformer models, e.g. Bidirectional Encoder Representations from Transformers (BERT) or Generative Pre-trained Transformer (GPT), have shown remarkable capabilities in understanding and generating human language. They have proven highly successful in NLP for tasks such as machine translation [2, 3], document summarization [4], document classification [5] and named entity recognition [6] or medical question answering [7].

In previous work, we identified eight categories of use cases of transformer models. They include documentation and clinical coding, workflow and healthcare services, knowledge management, interaction support, patient education, health management, public health monitoring, and decision support [8]. Mesko discussed hypothetical future scenarios for LLMs, including remote patient diagnosis and surgical training. He highlighted the potential benefits of multimodal LLMs, such as processing different content types, overcoming language barriers, supporting interoperability in hospitals, analyzing scientific data with sentiment and context awareness, and supporting privacy protection [9]. Li et al. introduced a transformer-based algorithm that predicts the likelihood of conditions in a patient’s future visit to a hospital based on data from the electronic health record [10]. Overall, transformer models have shown significant performance gains in medical problem summarization [11] and clinical coding [12].

In view of possible use cases and encouraging results from research, it is of high relevance to reflect in this early stage of the era of applying transformer models in healthcare on their potentials, risks and shortcomings. Such reflection is necessary for a responsible design of applications. It will help in developing sustainable and efficient solutions that make use of this technology and truly improve healthcare outcomes by minimizing the risks. The research objective of this paper is therefore to identify the potentials, shortcomings and risks associated with the use of transformer models in healthcare by conducting a qualitative study with 28 participants. Additionally, we aim to assess what is needed for considering applications based on such models reliable. This knowledge will help in developing solutions that will be accepted by their users. Furthermore, the results will enable us to establish a research agenda for the development of applications based on transformer models. To the best of our knowledge, this is the first study to explore the opinions of researchers in the field of health NLP on the use of transformer models in the health sector. We are aware of research papers envisioning the future landscape of LLMs in medicine [9, 13]. However, these papers only basically summarize ideas of their authors while we focus on conducting an online survey and a qualitative analysis and base our results on a broader expert basis. Other papers assessed the potentials and risks of ChatGPT as a health application in an experimental manner [14, 15]. We are focusing not on this commercial product that has not specifically developed for healthcare purposes, but on the potentials and risks of applying the technology in tailored applications.

## Methods

To achieve our goal, we conducted an online survey with qualitative analysis. It was distributed among researchers working in the field of NLP in healthcare. They were recruited via email from the IMIA Participatory Health and Social Media Working Group, the authors’ peer networks, or by contacting researchers who were listed as corresponding authors in papers on transformer models in healthcare. Participants were given a brief definition of transformer models to ensure that all considered the same definition and were imagining not only the currently popular OpenAI’s ChatGPT but also the underlying technology. The questionnaire included a series of demographic questions and 7 open-ended questions: (1) What are the benefits of transformer models in healthcare? (2) Which shortcomings of applying transformer models in healthcare do you see? Which risks do you see for the (3) medical profession, (4) patient care, (5) health IT, (6) data protection in regard to the adoption of transformer-based models in health IT?, (7) When would you consider digital solutions based on transformer models to be reliable?

The questionnaire was open for three weeks from 10 April to 1 May 2023. No reminders were sent. All responses to the open-ended questions were analyzed by the authors using a simplified thematic analysis [16]. After the survey was administered, two authors (KD, OR) independently read the responses, familiarized themselves with them and grouped the responses into categories. Categories were checked for consistency and simplicity (themes included all coded factors (inclusive) and two categories could not be assigned to one response (exclusive)). Finally, suitable names and definitions were created for each category. The final groups were formed in discussion between the two authors (KD, OR). Conflicts were discussed with a third author (RM). To report the results of the survey, considering size restrictions, we followed the Checklist for Reporting Results of Internet E-Surveys (CHERRIES) [17] and Consolidated criteria for Reporting Qualitative research (COREQ) checklist for qualitative studies [18]. A clarification of responsibility was submitted to the ethics committee of Cantone Berne who confirmed that no ethics approval is necessary for conducting the study as described before.

## Results

In this section, we summarize the demographics of the panel and the results of the thematic analysis. Quotes undermining the identified themes are available in Appendix 1.

### Delphi Participant Panel

The panel consisted of 28 researchers (25% female, *n* = 7). An exact response rate cannot be provided as we allowed the recruited participants to share the link to the survey with their network. Our estimated response rate is 26.4% since we directly contacted 44 persons and the IMIA Working group mailing list comprises 78 e-mail addresses. Basic demographics are summarized in Table 1. A total of 10.7% reported being experts in transformer models, 25% used their basic functions regularly, 28.6% knew how they work, and 32.1% tested OpenAI ChatGPT but had only basic knowledge of the underlying technology. One person had no knowledge of transformer models - we excluded this person’s response for reasons of validity.

**Table 1:** Demographics of the study participants

Background	Computer science / Engineering	Medicine	Nursing	Other health sciences	Other
	39.3%	28.6%	3.6%	10.7%	7.1%
**Professional experience**	**More than 10 years**	**5–10 years**	**Less than 5 years**		
	85.7%	3.6%	10.7%		
**Working sector**	**Academia**	**Public health sector**	**Privat health sector**		
	92.9%	17.9%	7.1%		
**Country of residence**	**Europe**	**Australia and Oceania**	**North America**		
	75%	10.7%	14.3%		

### Benefits of Transformer Models in Healthcare

**Fig. 1:**
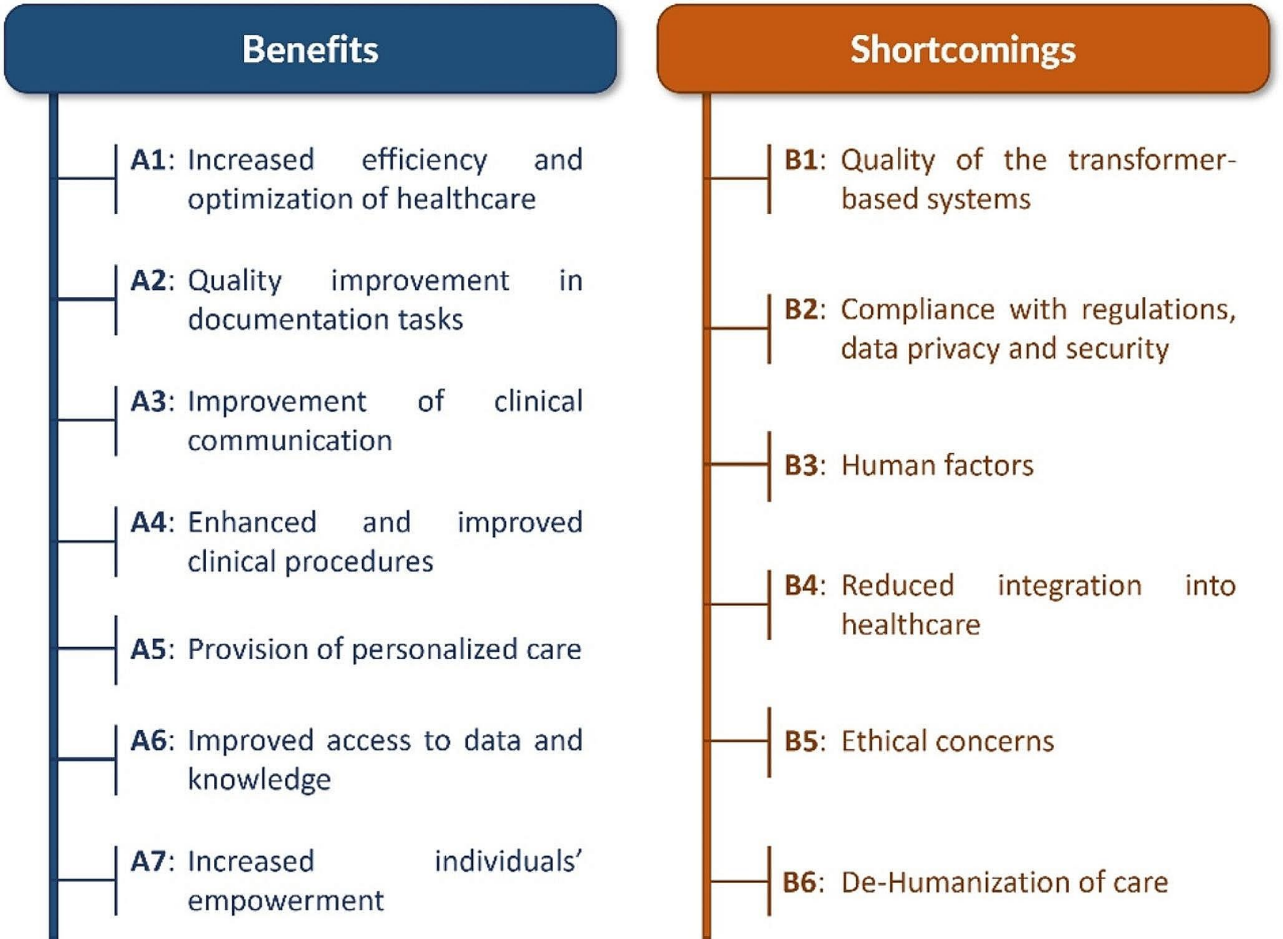
Identified benefits and shortcomings of the use of transformer models in healthcare

Seven themes were identified among the participants’ responses to the question regarding the potential of applying transformer models in healthcare applications (see Fig. 1):

- A1: Increased efficiency and optimization of healthcare.
  - Transformer models can improve healthcare efficiency by accelerating diagnoses and automating tasks like triage, appointment scheduling, and clinical trial matching. This automation helps reallocate human resources to critical tasks, reducing their burden and workload.
- A2: Quality improvement in documentation tasks.
  - Transformer models can improve clinical documentation by summarizing large amounts of information and tailoring the writing style for different readers, reducing the burden on healthcare professionals and improving documentation quality.
- A3: Improvement of clinical communication.
  - Transformer models can improve clinical communication between health professionals and with patients by reducing errors and tailoring information to the language, cultural level or age of the recipient. They could also facilitate the collection of information from patients at a distance during initial contact or follow-up.
- A4: Enhanced and improved clinical procedures.
  - Transformer models could improve healthcare processes through evidence-based decision making, accurate diagnoses through automated data analysis and prediction (e.g. “help in identifying patterns and predicting outcomes in healthcare data”), and automated generation of treatment plans (e.g. ”develop more effective treatment plans”).
- A5: Provision of personalized care.
  - Automatic data analysis using advanced algorithms enables the implementation of personalized medicine. In this regard, some participants pointed out that treatment and diagnosis can become personalized and preventive by transformer model-based systems.
- A6: Improved access to data and knowledge.
  - Transformer models improve data access and processing for better knowledge creation, efficiently extracting relevant information from large, unstructured healthcare data. They also enable easier human-computer interactions, such as voice user interfaces to access information and knowledge.
- A7: Increased individuals’ empowerment.
  - Transformer models in healthcare will empower individuals, patients, carers as well as health professionals, by supporting them through information provision and enhancing their knowledge as needed.

### Shortcomings of Transformer Models in Healthcare

Six themes were identified among participants’ responses to the question regarding the potential shortcomings of the use of transformer models in healthcare (see Fig. 1):

- B1: Quality of the transformer model-based systems.
  - This theme comprises two subthemes: system development aspects and erroneous system results. System development issues arise from data dependency, as the quality of transformer models is affected by biases in the training data, such as race and gender bias. Participants noted the need for high-quality, annotated data for training purposes, which is limited due to high annotation costs. The second subtheme, erroneous system results, involves risks from incorrect information provided by transformer models. Challenges include verifying information, dealing with errors or hallucinations and the lack of explainability and interpretability. These issues could harm patients and reduce health professionals’ trust and acceptance of these models. Participants emphasized the importance of testing transformer models in healthcare and real-world scenarios to ensure reliability.
- B2: Compliance with regulations, data privacy and security.
  - Transformer model-based systems must comply with privacy regulations and protect the privacy of sensitive health data, particularly from potential third-party access and misuse.
- B3: Human factors.
  - This theme relates to the health professionals who are expected to use systems based on transformer models. Issues include the need for human expertise to judge the results and their accuracy, overreliance, carelessness and the underdevelopment of skills.
- B4: Reduced integration into healthcare.
  - The theme concerns the reduced integration of transformer model-based systems into healthcare workflows and challenges related to their uptake and use. Participants identified the increased complexity of care caused by the proliferation of information, including that generated by transformer model-based systems, as a key challenge to adoption and use by healthcare professionals.
- B5: Ethical concerns.
  - Biased training data could exacerbate health inequalities, and the need for technical resources and professional training, which is not uniformly available across health centers, could further contribute to inequalities.
- B6: De-humanization of care.
  - Transformer models could affect the doctor-patient relationship by reducing interaction and increasing de-humanization. The automation of care processes could also make patients feel treated as numbers.

**Fig. 2:**
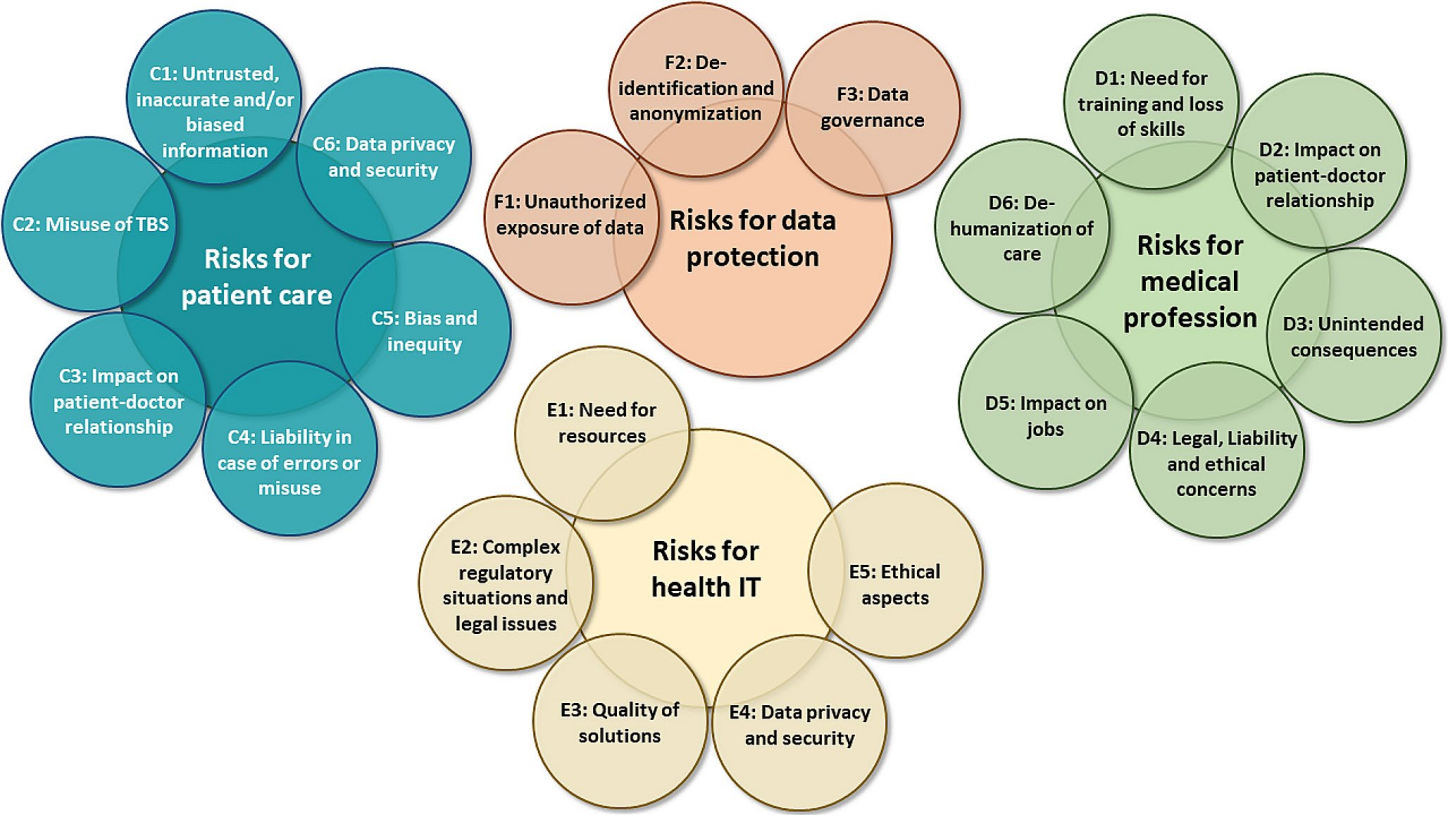
Identified risks of the use of transformer models in health

### Risks Associated with the Use of Transformer Models in Healthcare

We asked the participants to reflect on the risks of the use of transformer models in healthcare from different perspectives: risks for patient care, for the medical profession, for health IT and for data protection. The results are summarized in the following.

#### Risks for PatientCcare

We identified six categories of risks for patient care associated with the usage of transformer models in healthcare applications (see Fig. 2):

- C1: Untrusted, inaccurate or biased information.
  - When used to provide clinical decision support, transformer models may lack accuracy or require verification, leading to the risk of misdiagnosis or incorrect treatment. The increasing availability of such models could lead to the use of unreliable or untested systems by health professionals, patients or carers, potentially causing harm.
- C2: Misuse of transformer model-based systems.
  - A major concern was over-reliance on these systems by both patients and professionals, potentially undermining patients’ self-management and decision-making skills in the care process. To mitigate this, participants emphasized the need for patient education on responsible use and correct interpretation of results from transformer model-based systems.
- C3: Impact on the patient-doctor relationship.
  - The patient-doctor relationship, normally based on trust, empathy, respect and continuity, could be compromised by overreliance on diagnoses or treatment suggestions from digital systems. Some participants noted that the excessive focus on these digital technologies by healthcare professionals could lead to worsen interpersonal relationship with patients. Patients could negatively perceive this overreliance because they could feel that digital solutions are replacing doctors resulting in a de-humanization of the healthcare. One participant commented that this deterioration in relationships could even extend to the institutions, leading to patients underestimating and distrusting the healthcare system.
- C4: Liability in case of errors and misuse.
  - The issue of liability is a major concern in relation to the risk of misdiagnosis and mistreatment. In cases where systems malfunction or fail, determining responsibility remains an unresolved challenge.
- C5: Bias and inequity.
  - Systems based on transformer models, which are often trained on biased data, could exacerbate health inequalities. Factors such as low literacy, accessibility issues and socio-economic status provide barriers to patient use of these solutions.
- C6: Data privacy and security.
  - Participants identified privacy and security risks in patient care (e.g. data breaches or unauthorized access to data) and emphasized that personal health information, especially sensitive data, is protected by law and is essential for a trusting patient-doctor relationship. They agreed that the processing of patient data by transformer model-based systems could lead to violations of patient rights.

#### Risks for the Medical Profession

We identified several risks for the medical profession (see Fig. 2):

- D1: Need for training on new competences, and loss of skills.
  - This category concerns overconfidence, overreliance, undervaluation, the need for specific education and training for health professionals, and the erosion of clinical skills and confidence in quality. Participants stressed the importance of training professionals to understand and correctly use and interpret the results of these systems, not to overrely or undervalue their results, and highlighted concerns about confidence in their quality and effectiveness. Health professionals need to learn when to trust the system versus their own expertise. Finally, there is concern that reliance on these systems could undermine critical thinking skills.
- D2: Impact on the patient-doctor relationship.
  - The negative impact on the patient-doctor relationship is a key issue regarding the risks of using transformer models in medicine. Participants agreed that these systems could reduce patient-doctor communication, potentially leading to a loss of patient trust and weakening the patient-doctor relationship.
- D3: Unintended consequences.
  - The use of transformer models in healthcare can lead to unintended consequences, such as incorrect diagnoses and inappropriate treatment plans, often due to incorrect model outputs or an overestimation of the models’ capabilities.
- D4: Legal, liability and ethical concerns.
  - Participants identified and discussed potential legal and ethical issues in the use of transformer models in healthcare, including privacy, data security and patient autonomy. Concerns were also raised about the liability of healthcare professionals for errors or misuse of these systems.
- D5: Impact on jobs.
  - The introduction of transformer models in healthcare could have an impact on jobs: creating new roles, changing existing roles and possibly leading to job losses in medical professions.

#### Risks for Health IT

In the following, the identified risks for health IT are described (see Fig. 2).

- E1: Need for resources to develop and integrate transformer models in healthcare systems.
  - Participants highlighted the need for multiple resources to develop, deploy, integrate and maintain transformer models in healthcare. They found the integration of these systems into existing health IT infrastructures to be particularly challenging. Concerns included development, integration and operational costs, which could exacerbate inequalities due to financial constraints in healthcare institutions. Lack of reimbursement models and time constraints were also significant factors. The need for specialized human resources and expert development of these systems was emphasized, and the risk of their unavailability was noted. In addition, specific training was considered essential for the effective uptake and use of transformer model-based systems.
- E2: Complex regulatory situation and legal issues.
  - Complex regulations in different countries, such as medical device regulations, General Data Protection Regulation (GDPR) and Health Insurance Portability and Accountability Act (HIPAA), already pose risks to the health IT sector and even more regulation is needed. The adoption of transformer models in health IT raises issues around intellectual property, patents and licensing, potentially hindering collaboration, knowledge sharing and industry adoption, and increasing the risk of litigation. Despite their potential to advance medical research, diagnosis and treatment, challenges remain in the ownership and licensing of these models. In addition, determining liability and responsibility for misdiagnosis and mistreatment due to incorrect system outputs remains a pressing issue.
- E3: Quality of solutions.
  - Participants identified quality issues related to transformer models, including the quality of information, data, models, validation and evaluation. They emphasized the importance of the quality of system results, noting that inaccurate, inappropriate or confusing information could lead to unintended consequences. The quality of systems was linked to training data, with concerns about the use of models outside their training context. Despite recognizing the need for high quality systems to prevent patient harm, participants found it challenging to evaluate and validate transformer models due to the lack of standardized evaluation frameworks. They also noted that competitive pressures to develop and market new tools could compromise system quality.
- E4: Data privacy and security.
  - Transformer models handle large amounts of sensitive data, which contributes to associated security and cybersecurity risks.
- E5: Ethical aspects.
  - Participants reported ethical concerns related to the use, development, and training of transformer models as important factors to consider.

#### Risks for Data Protection

Participants’ answers to the question on risks related to data protection resulted in three categories of topics (see Fig. 2):

- F1: Unauthorized exposure of data.
  - The use of transformer models in healthcare could lead to confidentiality issues, including unauthorized data disclosure, breaches of privacy regulations, data leakage, and insecure data storage and transmission.
- F2: De-identification and anonymization.
  - Participants raised concerns about de-identification and anonymization in transformer models, noting the risk of exposing sensitive data and the use of weak anonymization techniques that reduce their trustworthiness.
- F3: Data governance.
  - There are risks of lack in transparency and a need for clear descriptions of how transformer model-based systems handle patient data. Concerns have also been raised about inadvertent disclosure of medical data to third parties during development, which poses privacy and security risks.

### Reliability of Health Systems Based upon Transformer Models

The free text answers to the question “When would you consider digital solutions based on transformer models to be reliable?” revealed three groups of aspects:

- G1: Supervised and transparent use.
  - Participants emphasized that the reliability of transformer model-based systems can increase when a human is involved. The ability to interpret and repeat results is key to reliability. The systems should explain how the model arrived at its results. Their use should be made transparent to patients.
- G2: Data integrity and generalizability.
  - Data quality, particularly in terms of diversity and representativeness of the target population and health context, was considered critical for reliability. Participants also identified generalisability as a key factor in the real-world applicability of transformer models.
- G3: System quality.
  - This theme covers aspects such as output, outcome, model quality, regulatory compliance, accuracy, efficiency, effectiveness, robustness, resilience, bias minimization and fairness. Key issues include compliance with security and privacy regulations, accuracy through validation and testing, and the importance of effectiveness and efficiency for reliability. Robustness and resilience of models are seen as critical, and minimizing bias and ensuring fairness are also essential for system reliability.

## Discussion

### Principal Results

This study examined opinions of researchers in the field of NLP in healthcare on the benefits, shortcomings and risks of applying transformer models in healthcare. Benefits include increased efficiency, process optimization, improved clinical documentation, better communication, automation of routine tasks and better decision making, as well as better data handling and patient empowerment. However, there are concerns about potential bias, auditability and privacy. Challenges include the need for expertise, ethical dilemmas and potential de-humanization of care. Specific risks for the medical profession include the impact on jobs, changes in the patient-doctor relationship, and the need for training in system use and data interpretation, with an anticipated loss of skills for both health professionals and patients.

### Relation to Other Work

Studies of NLP tasks using transformer models are consistent with participants’ views of potential improvements in documentation tasks. These models have shown promise in areas such as radiation oncology [19], medical problem summarization [11] and clinical coding [12], and offer potential for text summarization, efficient writing and multilingual communication [20]. This potential related to a positive impact on efficiency and optimization of healthcare tasks are supported by Thirunavikarasu et al., who concluded that “studies are needed to ensure that LLM tools actually reduce workload rather than introducing an even greater administrative burden for healthcare” [21]. Given the early stage of development of digital health solutions based on transformer models, there is little evidence from studies to show the efficiency gains achieved by such solutions. However, there are significant concerns about misinformation from LLMs, as highlighted by participants and researchers such as Eggmann et al. [20] and De Angelis et al. [22].

Re-identification was considered a significant risk by participants. However, they did not define potential differences among several contexts such as rare conditions. Shortcomings such as model quality, privacy, security, ethical issues and human factors are also recognized in the literature [23]. Reddy et al. proposed an evaluation framework for the application of LLMs in healthcare to address these risks [24].

We found dependencies between different aspects, such as system errors and liability. If transformer models produce wrong information and cause (wrong or unnecessary) patient treatment, this not only poses risks to patient care but also raises liability concerns and would have an economic impact. We argue that the “human in the loop” approach offers a valuable layer of supervision and verification that serves as a key link to mitigate these concerns. Ahmed et al. also argue for human involvement to validate the results of LLM-based systems and prevent patient harm [25].

Legal regulations, such as GDPR and HIPAA or ISO/IEC 27,000 series are of major importance to ensure the responsible use of applications in healthcare. Mesko and Topol argue in favor of a regulatory oversight that should assure medical professionals and patients can use transformer-model-based systems without causing harm or compromising their data or privacy [26]. Their practical recommendations include creating a “new regulatory category for LLMs as those are distinctively different from AI-based medical technologies that have gone through regulation already”. However, it is also worth discussing the balance between regulation and innovation. Finding a proper balance is important (albeit highly complex) to promote the adequate development and deployment of new technologies while maintaining the trust and privacy of patients. To avoid hampering innovation we recommend a responsible design and development, that includes reflections of possible risks in the early stages of solution design. Several tools supporting this issue have been developed recently, e.g., the risk assessment canvas for digital therapeutics [27] or the digital ethics canvas [28]. In addition, Harrer proposed a comprehensive framework for the responsible design, development and use of LLM-based systems [29]. This framework focuses on ensuring fairness, accountability, privacy, transparency, accountability and alignment with values and purposes, reflecting key aspects identified in the survey. This approach emphasizes the need for careful consideration of ethical, technical and cultural issues in the development and use of LLMs in healthcare.

Additionally, efforts are underway to address biases in transformer models, as exemplified by Mittermaier et al.‘s strategy for mitigating bias in surgical AI systems [30]. These initiatives are critical to improving the accuracy and fairness of healthcare supported by transformer model-based systems [30]. The proliferation of digital health has enabled the elimination of certain barriers in healthcare by reducing disparity. However, the use of these technologies has led to the emergence of new factors affecting health equity. Despite being a highly relevant topic, participants did not mention any specific health disparity considerations. There is an urgent need for standardized evaluation frameworks, evaluation standards and metrics to ensure that these models meet essential requirements such as accuracy, effectiveness and reliability. This is in line with the work of Guo et al., who highlight that LLMs can potentially leak private data or produce inappropriate, harmful or misleading content [31]. Guo et al. acknowledged the importance of evaluating LLMs from multiple perspectives, including knowledge and skills, alignment, and security [31]. The risk of dehumanization can also be controversial: Dehumanization could have a positive impact on patient care by reducing the shame that occurs in human-to-human communication, thereby better promoting and protecting important medical values [32].

### Research Agenda

As indicated at the beginning, one objective of this study was to derive a research agenda for the development of applications based on transformer models in healthcare. For successful real-world application, a comprehensive approach is necessary, including:

- Responsible design: Considering ethical and other risks during development to create solutions that mitigate these issues.
- Utilizing real-world data: Evaluating model quality and performance using authentic, diverse healthcare data for a realistic assessment of capabilities.
- Testing and Integration: Rigorous testing and seamless integration into health IT systems and workflows to ensure practicality and effectiveness in clinical settings.
- Education and training: Providing education and training for patients and health professionals to improve interaction with transformer-based systems [33].
- Continuous risk assessment: Ongoing evaluation of potential risks and shortcomings during the design and development process.
- Postmonitoring procedures: Implementing robust postmarketing surveillance to ensure patient safety, quality, transparency, and ethics, addressing challenges and risks over time [34].

### Limitations

The study’s participants, mainly from computer science, health informatics and medicine, were predominantly affiliated with academic institutions, mainly in Europe. This skewed representation, with only a third coming from regions such as Australia/Oceania and North America, may affect the applicability of the study, especially given Europe’s established healthcare systems and strict privacy regulations. This demographic imbalance could limit the relevance of the findings in areas without similar regulatory, economic and infrastructural contexts, impacting on the adoption and use of the transformer model. In addition, while most participants had experience in health informatics, only about a third had specific experience with transformer models, mostly limited to testing OpenAI’s ChatGPT. This lack of extensive knowledge of transformer models could affect the reliability of their assessments. The selection of participants based on publication records and involvement in a working group introduced a selection bias. To reduce bias in the thematic analysis, it was conducted by two independent people.

Another limitation of this study is that the user responses were sometimes not comprehensive enough to extract sufficient detail. Therefore, some of the items listed before remain vague. For example no specific aspects were mentioned where professionals would need training (item B5). Data privacy and security issues were identified as potential risks of using LLMs in healthcare. Some examples of the potential risks were mentioned but deeper analysis should be done in further research. As it is a qualitative study with time limitations, some themes were not addressed in depth.

## Conclusions

Transformer models and LLMs have the power to transform healthcare systems and processes. They offer remarkable advances in diagnosis, treatment, communication, clinical documentation and workflow management. These models contribute to personalized care, increase patient empowerment, and improve access to data and medical knowledge. However, these technologies also pose various risks and limitations, which can be broadly classified into three categories: data-related issues, system use and its impact, and system quality and regulatory concerns. From an economic perspective, there is a need to establish training programmes and a potential shift in the employment landscape within the healthcare sector.

A number of considerations are critical to the reliable application of these models:

- Human-in-the-loop systems to ensure oversight and accountability.
- Transparency in explaining the results of these models.
- Ensuring high quality data.
- Maintaining robust system quality, including reliability and accuracy.
- Compliance with regulatory standards.

In summary, the integration of transformer models in healthcare offers significant potential for innovation and improvement. However, it requires a careful and multi-faceted strategy to ensure its safe and effective implementation. By following these considerations for reliable applications, we can harness the transformative power of these technologies while maintaining the highest standards of patient care and well-being in the dynamically evolving healthcare technology landscape.

**Appendix 1: Quotes of the participants’ responses for the identified themes**.

## Electronic Supplementary Material

Below is the link to the electronic supplementary material.

Supplementary Material 1

## Data Availability

No datasets were generated or analysed during the current study.
