# Supplementary material for: Transformer Models in Healthcare: A Survey and Thematic Analysis of Potentials, Shortcomings and Risks
Source: J Med Syst. 2024 Feb 17;48(1):23. doi: 10.1007/s10916-024-02043-5 (PMC10874304; doi:10.1007/s10916-024-02043-5)
Supplement: Supplementary file 1 — Supplementary Material 1 [file 10916_2024_2043_MOESM1_ESM.docx]

Appendix 1: Quotes of the experts’ responses for the identified themes

| **Theme** | **Quotes** |
| --- | --- |
| **Benefits of transformer models in healthcare** | |
| A1: Increased efficiency and optimization of healthcare | *“Improving process automatization, reducing workload, … , allowing redistribution of healthcare staff on priority tasks leading to improved health services by reduced burden on routine tasks” P1*  *“Transformer models can analyze patient symptoms and help identify potential health issues more quickly, speeding up the diagnostic process and facilitating timely treatment.” P3*  *“By automating many manual tasks, such as data extraction and analysis, transformer models can help healthcare providers to improve their workflow and increase efficiency.” P7* |
| A2: Quality improvement in documentation tasks | “*make it easier for healthcare professionals to maintain accurate records of their interactions with patients*” *P3* |
| A3: Improvement of clinical communication | *“By providing patients with virtual health assistants, transformer models can offer a more accessible and convenient way for patients to obtain health information and address their concerns.” P3*  *“… very good tools for *verbalising* interactions: ie. they take care of the dialogue end of things, while the actual (and factual) knowledge and data comes from elsewhere (either humans or other online sources)” P6*  *“Patient-Doctors interaction could benefit from the use of natural language, making it easier to understand symptoms, questions and instructions. Furthermore, this interaction can be made in different languages, or even in the same language but adapting the expressions to the cultural level or age of the patient.” P14* |
| A4: Enhanced and improved clinical procedures | *“Transformer models can analyze patient data to make predictions about the presence of specific conditions, helping healthcare providers to diagnose diseases more accurately and develop more effective treatment plans” P7*  *“Transformer models have shown to have high accuracy and efficiency in processing natural language and medical images, which can improve the speed and accuracy of medical diagnosis and treatment. They can also help in identifying patterns and predicting outcomes in healthcare data.” P24* |
| A5: Provision of personalized care | “*Transformer models can be used to build predictive models that can identify patients at risk of certain conditions, allowing for earlier interventions and more personalized care*.” *P7* |
| A6: Improved access to data and knowledge | “*Transformer models can process large amounts of unstructured medical data, such as electronic health records (EHRs), and extract relevant information to support decision-making.” P7*  *“Ability to understand and interpret large quantities of unstructured healthcare data” P9*  “*Enabled voice user interface that is natural and intuitive*” *P9* |
| A7: Increased individuals’ empowerment | *“… increased patients and caregivers' empowerment; engaging education for healthcare professionals…” P11* |
| **Shortcomings of transformer models in healthcare** | |
| B1: Quality of the transformer model-based systems | **System development aspects**  *“The performance of transformer models is only as good as the quality and diversity of the data they are trained on. In healthcare, there may be issues with data quality, including missing or incorrect information, and bias in the data that can impact the performance of the models.” P4*  *“There is also a risk of bias and errors in the model if the data used for training is not diverse and representative or contains some annotation bias or error. For example, in medical information extraction, the annotation of entities and relations require much effort from health professionals and they sometimes disagree with each other on specific cases. Annotating more training samples could increase the diversity of data but also significantly increase the cost. Effective sample selection would also be needed to choose more representative data samples for annotation“ P21*  *“hallucination, not factual answers but probabilistically plausible answers … impossible to find out degrees of reliability of the answers … impossible to trace answers back to original sources” P3*  *“One of the challenges of using deep learning models, such as transformer models, is that they can be difficult to interpret and understand, making it challenging to assess their performance and trust their outputs” P4*  **Incorrect system results**  *“Need to be trusworthy and explainable AI. If clinical staff does not understand why decisions are taken (when dealing about health care decisions), they will not use it.” P13*  *“Testing and training of these models in healthcare environment is mandatory” P14* |
| B2: Compliance with regulations, data privacy and security | *“The use of transformer models in healthcare is subject to strict regulations, such as the Health Insurance Portability and Accountability Act (HIPAA) in the US, and it is important to ensure that these models are developed and used in compliance with relevant regulations.” P4*  *“Health data is highly sensitive and protected by privacy laws, and the use of transformer models in healthcare requires proper handling of this sensitive information to ensure patient privacy and data security.” P4* |
| B3: Human factors | *“While transformer models can automate many tasks, they still require human expertise to ensure that their outputs are accurate and relevant. Healthcare providers must be trained to use these models effectively and interpret their outputs” P4*  *“The risk exists that clinicians will let several erroneous plans 'slip through the cracks' because they are not creating the plans/communications from scratch themselves.“ P24*  *“By automating various tasks, clinicians have less 'hands on experience' for certain tasks, and may therefore encounter issues when the models are inaccessible or incorrect.”. P24* |
| B4: Reduced integration into healthcare | *“Having too much information may confuse them” P9* |
| B5: Ethical concerns | *“… potential health inequities because of lack of competence to use these tools” P8*  *“The implementation of AI in healthcare raises various ethical questions, including issues related to fairness, transparency, and accountability. For instance, biased algorithms can inadvertently perpetuate existing healthcare disparities, disadvantaging certain patient populations.” P3* |
| B6: De-humanization of care | *“Potentially, automation can give patients the idea that they are not seen as a human, but rather as a bunch of numbers that gets put into a machine” P20* |
| **Risks for patient care** | |
| C1: Untrusted, inaccurate and/or biased information | **Misdiagnosis or incorrect treatment**  *“Misdiagnosis and incorrect treatment: Inaccurate or misleading information provided by transformer-based models can lead to misdiagnoses or inappropriate treatment recommendations, which could negatively impact patient care and even cause harm.” P3*  *“Incorrect Diagnoses and Treatment Plans: There is a risk that transformer-based models may produce incorrect diagnoses or inappropriate treatment plans, leading to harm to patients. It is important to thoroughly test and validate these models to minimize this risk.” P7*  **Unverified models causing harm**  *“The use of unverified versions direct to patient care may cause risk on personal health decisions especially in mental health domain and pediatric settings.” P1*  *“The problem could arise from the proliferation of models from different sources and the patient consulting unreliable models.” P12* |
| C2: Misuse of transformer model-based systems | **Overinterpretation**  *“Over-reading of images leading to unnecessary additional tests.” P4*  **Overreliance**  *“Over-reliance on these tools because they sound very convincing”. P4*  *“Overreliance on Technology: There is a risk that medical professionals may become overly reliant on the outputs of transformer-based models, leading to reduced critical thinking and decision-making skills” P7*  **Loss of self-management and decision-making skills**  *“increased dependency on technology: patients may trust the medical advice of the transformer model more than their physician or even their own judgement which may lead to a lack of autonomy and reduced empowerment for decision making”. P15*  **Missing education**  *“Patient education in the limits of these tools and issues associated with the quality of the results and contents derived from these tools”. P18* |
| C3: Impact on the patient-doctor relationship | **Loss of contact between patient and health professional**  *“May not seek medical attention based on inaccurate diagnosis by using chat GPT” P13*  *“Replacement of health care professional's decision with model-generated decision”. P22*  **Mistrust in healthcare system**  *“Negative feelings because they can perceive that IT are replacing doctors” P11*  *“De-humanization of care: Potentially, automation can give patients the idea that they are not seen as a human, but rather as a bunch of numbers that gets put into a machine.” P23* |
| C4: Liability in case of errors and misuse |  |
| C5: Bias and inequity | **Exacerbate existing health disparities and inequities**  *“Bias and inequality: If transformer-based models are trained on unrepresentative or biased data, they might perpetuate or exacerbate existing biases in healthcare. This could result in unfair treatment for specific patient populations, exacerbating health disparities.” P3*  *“Bias in the Models: If the training data used to develop transformer-based models is biased, the models may produce biased outputs that disadvantage certain patient populations. It is important to ensure that the training data is diverse and representative to avoid this risk.” P7*  *“suffering discrimination due to the tool's biases (in minorities or rare diseases, for example)” P14*  **Limited access to the technology increasing inequity**  *“The technology might not be accessible to all patients which may have an impact on health equity”. P15* |
| C6: Data privacy and security | *“Data privacy and security: The use of transformer-based models in health IT involves handling sensitive patient data. Data breaches or unauthorized access to this information can compromise patient privacy, erode trust in healthcare providers, and have legal implications.” P3* |
| **Risks for the medical profession** | |
| D1: Need for training on new competences and loss of skills | **Overreliance and undervalidation**  *“Overconfidence and under-validation when it comes to use such feedback mechanisms in clinical decisions.” P1*  *“The use of such models can lead to overreliance and to a reduction in human decision making. this is problematic when results are biased or the automatic decision-making process is not reproducible.” P12*  **Difficult interpretation of results**  *“too much or not enough trust - it will take time to determine what to trust; how do we fit them into workflow - we need a lot of research on optimising workflow” P2*  *“Need to increase their knowledge to understand the basics of the underlying systems that might be in place in the future.” P15*  *“The medical professions need to be careful and think critically about the model’s prediction”. P19*  *“Difficulty in interpretation of results and connection to practically relevant user scenarios in terms of assistance to clinical practice for evidence based medicine”. P2*  **Loss of competencies**  *“Over-reliance on AI tools may lead to the erosion of essential clinical skills and decision-making abilities, as healthcare professionals might rely too heavily on the models' outputs without critically assessing the situation.”* |
| D2: Impact on patient-doctor relationship | *“The use of transformer models may reduce the amount of direct patient-provider interaction” P12*  *“Patients trusting AI more than their clinicians.” P16*  *“degradation of the patient-provider relationship” P17* |
| D3: Unintended consequences | **Incorrect diagnosis or treatment**  *“The use of transformer-based models in healthcare can have unintended consequences, such as incorrect diagnoses or inappropriate treatment plans. It is important to thoroughly test and validate these models before they are widely adopted to minimize these risks.”. P4*  *“If a transformer-based model provides inaccurate or misleading information, it could lead to misdiagnosis or incorrect treatment recommendations, potentially causing harm to patients”*  **Overestimation of system capabilities**  *“Temptation from non-health professionals to replace health practitioners with some of these tools.”.P15* |
| D4: Legal, liability and ethical concerns | **Privacy, data security, patient autonomy at risk**  *“The use of transformer-based models in healthcare raises a number of legal and ethical concerns, such as privacy, data security, and patient autonomy. It is important to ensure that these models are developed and used in compliance with relevant regulations and ethical guidelines.” P4*  **Liability for errors**  *“Determining liability in cases involving AI errors could be challenging, raising questions about who is responsible for any negative outcomes— the healthcare professional, the AI system, or the technology provider”.* |
| D5: Impact on jobs | *“The use of transformer-based models in healthcare has the potential to automate many tasks, leading to job loss for some medical professionals. It is important to consider the potential impact on employment and ensure that affected workers are provided with training and support to transition to new roles.” P4*  *“New types of jobs or skills will be needed. Some tasks might become obsolete.” P12* |
| **Risks for health IT** | |
| E1: Need for resources to develop and integrate transformer models in healthcare systems | **Integration into current health IT infrastructure**  *“Their development and maintenance require significant resources, including technical expertise, computing power, and data storage. … Challenges in integrating these models with existing health IT infrastructure and ensuring that they are interoperable with other systems.” P15*  **Financial aspects of reimbursement**  *“Health IT research and industry organizations may face financial and resource constraints that limit their ability to invest in the development, implementation, and maintenance of transformer-based models. This can slow down innovation, create disparities in access to advanced AI technologies, and hinder the overall progress of the field.” P3*  *“The development and deployment of transformer-based models in healthcare can be expensive, requiring significant investment in technology and infrastructure. There is a risk that these costs may be prohibitive for some organizations, limiting the adoption and use of these models.” P7*  **Need for specific training**  *“The development and deployment of transformer-based models in healthcare can be technically challenging, requiring specialized knowledge and expertise. There is a risk that the field may not have the necessary resources and talent to effectively develop and use these models.” P7*  *“need of expertise and education may reduce the level of utility and adoption” P1* |
| E2: Complex regulatory situation and legal issues | **Need for regulation**  *“The use of transformer-based models in healthcare is subject to strict regulations, such as the Health Insurance Portability and Accountability Act (HIPAA) in the US, and there is a risk that organizations may not have the resources and expertise to ensure compliance with these regulations.” P7*  *“The field of heath IT should be much more regulated to ensure the reliability and security of the services provided and the correct processing of the data obtained. “ P14*  **Ownership and licensing**  *“The adoption of transformer-based models in health IT can raise issues around intellectual property rights, patents, and licensing. These concerns can create barriers for collaboration, knowledge sharing, and industry adoption, as well as contribute to potential legal disputes.” P3*  **Liability**  *“responsibility of misdiagnosis or incorrect treatment due models’ inaccurate prediction”.* |
| E3: Quality of solutions | **Unintended consequences**  *“these models can provide confusing information that seems to be provided by health professionals”.*  **Quality of training data**  *“The performance of transformer-based models is only as good as the quality and diversity of the data they are trained on. There may be challenges in obtaining high-quality and diverse health data to train these models, leading to lower performance and accuracy.” P7*  *“Health IT workers should be crucially aware of the intricacies of medicine and the areas of potential bias. When model developers are not aware of these issues, the model can be severely flawed”. P22*  **Missing evaluation standards**  *“Health IT research and industry may lack standardized approaches to validate and evaluate transformer-based models, resulting in models that are not rigorously tested or scrutinized. This could lead to the use of models that do not meet necessary safety, efficacy, and reliability requirements.”. P3*  **Low quality systems due to competitive pressure**  *“The rapid evolution of AI technologies, including transformer-based models, can lead to competitive pressures that drive premature or hasty adoption of models in health IT without appropriate due diligence, potentially putting patient safety and privacy at risk.” P3* |
| E4: Data privacy and security | *“Security levels against cyber-attacks must be maximized.” P14* |
| E5: Ethical aspects | *“Ethical issues and possible bias when these models have been trained with data from other contexts.” P16* |
| **Risks for data protection** | |
| F1: Unauthorized exposure of data | *“The use of models may depend on cloud services from third party service providers which require health institutions to ensure the services are compliant with regulations, especially HIPAA. Going with unregulated or non-compliant services may risk for data breach, privacy, and security” or “There is a risk that patient data may be accessed or used inappropriately, leading to breaches of privacy and confidentiality.” P1* |
| F2: De-identification and anonymization | *“Transformer models may inadvertently memorize or expose sensitive patient information during training or inference. If these models reveal identifiable patient data, it could violate data protection regulations and compromise patient confidentiality. … Properly de-identifying and anonymizing healthcare data is critical for privacy-preserving AI applications. However, the complex nature of healthcare data and potential correlations between variables can make it challenging to achieve effective de-identification, posing a risk to patient privacy.” P3* |
| F3: Data governance | **Lack of transparency**  *“The inner workings of transformer-based models can be difficult to understand, making it challenging to assess their performance and trust their outputs. This lack of transparency can make it difficult to ensure that patient data is being used appropriately and in compliance with relevant regulations”. P7*  **Undesired data sharing with third parties**  *“In the process of developing and deploying transformer-based models, healthcare data may be shared with or accessed by third-party partners or vendors, raising concerns about data security, accountability, and control over the use and handling of sensitive data.” P3* |
| **Reliability** | |
| G1: Supervised and transparent use. | **Human in the loop**  *“When there is a control mechanism or human in the loop, it may improve reliability of the solutions.” P1*  *“Transformer models should be transparent and explainable so that healthcare professionals and patients can understand how the model arrived at its results” P15*  *“The ability to understand and explain the reasoning behind model predictions and recommendations is essential for trust and usability in healthcare. Reliable transformer models should provide insights into their decision-making process, allowing healthcare professionals to validate and interpret the outputs.” P3*  **Repeatability**  *“The models should produce consistent and repeatable outputs, allowing for reliable and trustworthy results over time” P7* |
| G2: Data integrity and generalizability | **Data diversity and representativeness**  *“The models should be trained on high-quality, diverse, and representative data to ensure that they perform well and do not introduce bias into their outputs.” P7*  *“The performance of transformer-based models is only as good as the quality and accuracy of the data they are trained on, and there is a risk that errors or inaccuracies in the data may lead to incorrect diagnoses or inappropriate treatment plans” P7*  **Generalizability**  *“Reliable transformer models should perform well across different patient populations, disease conditions, healthcare settings, and data sources. This ensures that the models are adaptable and effective for a wide range of real-world scenarios.” P3* |
| G3: System quality | **Compliance with regulations**  *“The models should be developed and used in compliance with relevant regulations, such as privacy and data protection laws, to ensure that they do not pose risks to the security and privacy of patient data.” P7*  **Accuracy of system outputs**  *“The outputs of the models should be accurate, reflecting the input data and producing relevant and trustworthy results.” P7*  **Effectiveness and efficiency**  *“The models should be validated in real-world clinical settings to assess their impact on patient outcomes, safety, and healthcare delivery. Evidence of their effectiveness in improving clinical decision-making or patient care is crucial for establishing reliability”. P3*  **Robustness and resilience of models**  *“The models should be robust and capable of handling a wide range of inputs, including outliers and edge cases, without producing errors or unintended consequences.” P7*  *“Reliable models should be resistant to adversarial attacks, noise, and data anomalies. They should maintain stable performance even in the presence of unexpected or out-of-distribution inputs.” P3*  **Minimizing bias**  *“Reliable models should minimize biases and ensure fair treatment for all patients, irrespective of their demographic or clinical characteristics. This requires diligent assessment and mitigation of potential biases in training data and model predictions.” P3* |
